# Mortality and Years of Life Lost Due to Occupational Injury in Iran (2012-2016)

**Published:** 2019-06-01

**Authors:** Mohammad Aghaali, Seyed Davood Mirtorabi, Mohammad Reza Ghadirzadeh, Seyed Saeed Hashemi-Nazari

**Affiliations:** ^1^Department of Epidemiology, School of Medicine, Qom University of Medical Sciences, Qom, Iran; ^2^Department of Addiction Studies, School of Advanced Technologies in Medicine, Tehran University of Medical Sciences, Tehran, Iran; ^3^Legal Medicine Research Center, Legal Medicine Organization, Tehran, Iran; ^4^Safety Promotion and Injury Prevention Research Center, Department of Epidemiology, School of Public Health and Safety, Shahid Beheshti University of Medical Sciences, Tehran, Iran

**Keywords:** Occupational injuries, Occupational health, Accidents, Occupational, Iran

## Abstract

**Background:** Occupational injury is a cause of premature mortality, mainly in low- and middle-income countries. Occupational injuries estimated to kill more than 300,000 workers worldwide every year. We estimated the years of life lost (YLL) of fatal unintentional occupational injuries in Iran for the five years of 2012-2016.

**Study design:** A cross-sectional study.

**Methods:** To estimate the YLL, registered deaths due to occupational unintentional injury were identified from the Iranian Legal Medicine Organization. Estimated YLL was calculated according to Global Burden of Disease 2010 guideline. Population life expectancy in each corresponding years was retrieved from the national health database. All data collected entered into Excel software for calculations.

**Results:** In 2012-2016, fatal unintentional occupational injuries were the cause of 8,606 deaths in Iran, resulting in 4.6 prematurely lost life yr per 1000 males and 0.3 yr per 1000 females among workers, every year. 98.7% of the deaths occurred in males. Males from 15 to 19 yr of age and females from 10 to 14 yr of age showed the highest YLL rates. The rate of YLLs per 1,000 workers per year was 3.99 overall, 4.6 in males, and 0.4 in females.

**Conclusion:** Premature mortality due to occupational injury is still a serious problem in the Iranian population. Our findings may be useful from a health policy perspective for designing and prioritizing interventions focused on the prevention of premature loss of life. Known prevention strategies need to be implemented widely to diminish avoidable injuries in the workplace.

## Introduction

Occupational injuries are among the most serious preventable health problems around the world. Since occupational injuries cause mortality and disability during the individuals’ productive periods, they have serious economic and social impacts^1^. In 2016, occupational injuries estimated to kill about 328,000 workers^2^.

Although occupational accidents in developed countries is declining, globalization has increased occupational accidents in developing countries^3^ mortality due to injuries tends to be higher in developing countries, where workers experience a greater number and variety of hazards that lead to injury^4^.

Because of these catastrophic outcomes, scientific studies are needed in order to develop promotional and preventive activities^5^. Unfortunately, in developing countries, there are not adequate data about distribution and related factors of occupational Injuries^4^.

Previous studies on occupational injury in Iran have used Iranian Social Security Organization database^1,6,7^ or Ministry of Labor and Social Affairs’ offices database^8^. Both databases include only insured populations, because of under-estimation, these data are not so reliable^6^. Mortality can be greater in uninsured populations^9^ and more than 50% of the workers in the world do not have coverage by insurance or social security^10^.

The working environment should be free of injury risk or disease risk factor but many thousands of workers worldwide remain exposed to hazardous substances both in the developed and developing countries^11^.

In this study, we estimated the years of life lost (YLL) of fatal unintentional occupational injuries in Iran for the five years of 2012-2016, based on Iranian Legal Medicine Organization (ILMO) database. This database includes insured and uninsured populations.

## Methods

### Data sources

All registered deaths due to occupational unintentional injury between Mar 20, 2012 (the beginning of the Iranian New Year) and Mar 21, 2017, were identified from IMLO.

In Iran, according to the law, suspicious deaths due to occupational accidents are referred to as the IMLO. These items are recorded in a separate form (Form No. 10), in which occupational injuries are classified into poisonings, fires, firearm, deflagration, falls, cold weapon, motor vehicle injuries, drowning, exposure to electric current, radiation, extreme ambient air temperature, and accidental exposure to other unspecified factors.

In this study, deaths due to unintentional occupational injuries were defined as any potentially avoidable death due to an external cause resulting from an exposure related to the person’s work^4^. Death during commuting to or from the workplace and death occurs out of Iran were excluded. Immigrant workers were included.

### Statistical analysis

To estimate YLL rates, total mid-year population of workers used as its denominator. Moreover, YLL rate was estimated based on economically active population (EAP). The total number of workers and EAP, by sex and age, estimated based on Iran National Population and Housing Census in 2011 and 2016^12^.

The economically active population includes all people aged 10 yr old and over who, in the week before the survey, participated in the definition of work, in the production of goods and services (employed), or in the ability to contribute (unemployed)^13^.

Estimated YLL was calculated according to Global Burden of Disease 2010 guideline. YLLs, are calculated as follows: YLL=Number of deaths * life expectancy at the age of death. Population life expectancy in each corresponding year was retrieved from the Global Burden of Disease 2010. We estimated 95% confidence intervals for the YLL by using bootstrapping with 10,000 resamples. All data collected entered into Excel software and performed calculations.

## Results

**Figure 1:**
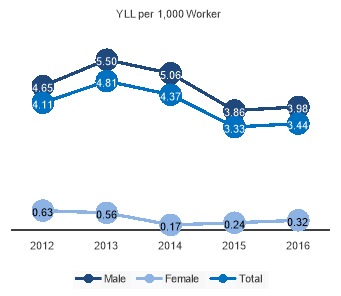
Year of life lost (YLL) rate of Fatal Occupational Injury per 1,000 Worker, in Iran

In 2012-2016, fatal unintentional occupational injuries were the cause of 8,606 deaths in Iran. Figure 1 shows the trend of mortality during study period. In women, the YLL of occupational injuries has declined from 2012 to 2014, but afterward it shows a rising trend. In men, we had two increases in YLL rates in 2013 and 2016.

The Mean±SD age of victims was 36.09±13.6 yr. 98.7% of the deaths occurred in males. Table 1 shows the distribution of victims. Most of them were 25 to 29 yr old. Table 2 shows the distribution of fatal occupational accidents by age groups.

**Table 1:** Distribution of variable among victims of Fatal Occupational injury in Iran during 2012-2016

**Variables**	**Frequency**	**Percent**
Sex		
Male	8,491	98.7
Female	115	1.3
Season		
Spring	2,055	23.9
Summer	2,731	31.7
Autumn	2,087	24.3
Winter	1,733	20.1
Cause of death		
Falls	3,858	44.8
Struck-By	1,868	21.7
Electric Shock	1,272	14.8
Asphyxiation	439	5.1
Fires	427	5.0
Drowning	131	1.5
Deflagration	115	1.3
Poisonings	37	0.4
Firearm	15	0.2
Other	444	5.2

**Table 2:** Fatal occupational injury in Iran by age groups

**Age group(yr)**	**Frequency**	**Percent**	**Cumulative Percent**
5-9	12	0.1	0.1
10-14	33	0.4	0.5
15-19	566	6.6	7.1
20-24	1282	14.9	22.0
25-29	1387	16.1	38.1
30-34	1224	14.2	52.3
35-39	1030	12.0	64.3
40-44	857	10.0	74.3
45-49	678	7.9	82.1
50-54	544	6.3	88.5
55-59	434	5.0	93.5
60-64	276	3.2	96.7
65-69	129	1.5	98.2
70-74	74	0.9	99.1
75-79	55	0.6	99.7
80+	25	0.3	100.0

**Figure 2:**
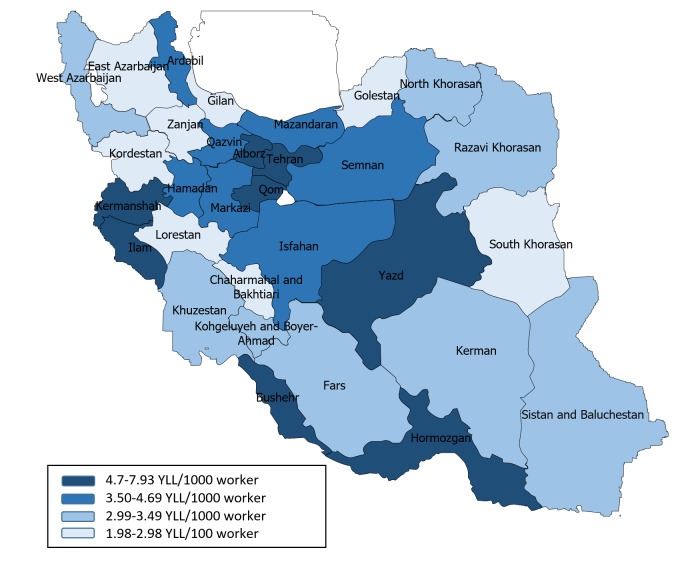
The rate of year of life lost (YLL) due to fatal occupational injury in 1,000 worker in Iran provinces (2012-2016)

The five provinces with highest YLL due to occupational injuries was found in the Yazd, Hormozgan, Alborz, Qom, and, Kermanshah respectively. Lorestan (1.98 YLL/1000 workers), Chaharmahal and Bakhtiari (2.37 YLL/1000 worker) and South Khorasan (2.42 YLL/1000 worker) had the lowest YLL. Rate of YLL due to fatal occupational injury in 1,000 workers in Iran provinces showed in Figure 2.

The rate of fatal occupational injuries per 100,000 workers per year was 7.9 overall, 9.09 in males, and 0.74 in females. In addition, the rate of fatal occupational injuries per 100,000 EAP per year was 6.83 overall, 8.02 in males, and 0.57 in females.

During five years, 433,251 yr prematurely lost due to fatal occupational injury in Iran. Males from 15 to 19 and females from 10 to 14 yr of age showed the highest YLL rates. The rate of YLLs per 1,000 workers per year was 3.99 (95%CI 3.72-4.28) overall, 4.6 (95%CI 4.31-4.9) in males, and 0.4 (95%CI 0.17-0.54) in females. Table 3 provides the YLL of occupational unintentional injury mortality by age group, and sex, illustrating that the mortality burden differs by age and sex.

**Table 3:** Year of life lost (YLL) of fatal occupational injury in Iran by age groups during 2012-2016

**Age group (yr)**	**Male**			**Female**			**Total**		
	**YLL**	**YLL per 1000 worker**	**YLL per 1000 EAP**	**YLL**	**YLL per 1000 worker**	**YLL per 1000 EAP**	**YLL**	**YLL per 1000 worker**	**YLL per 1000 EAP**
≤14	3,199	12.8	10.5	1,115	13.8	11.5	4,314	13.07	10.77
15-19	37,874	15.1	11.9	1,294	3.0	1.9	39,169	13.32	10.11
20-24	81,147	11.2	7.8	1,193	1.1	0.5	82,341	9.86	6.53
25-29	80,272	5.6	4.5	366	0.1	0.1	80,638	4.71	3.61
30-34	64,973	4.0	3.6	658	0.2	0.2	65,630	3.43	2.97
35-39	50,646	3.6	3.4	219	0.1	0.1	50,864	3.07	2.81
40-44	38,253	3.3	3.1	394	0.2	0.2	38,647	2.87	2.71
45-49	26,774	2.8	2.7	142	0.1	0.1	26,915	2.46	2.36
50-54	19,069	2.8	2.7	191	0.2	0.2	19,259	2.49	2.39
55-59	13,190	2.7	2.6	83	0.2	0.2	13,273	2.49	2.40
60-64	7,190	2.7	2.6	21	0.1	0.1	7,210	2.37	2.29
>65	4,879	1.7	1.7	113	0.2	0.2	4,991	1.44	1.41
Total	427,464	4.6	4.1	5,787	0.4	0.3	433,251	3.99	3.45

EAP: economically active population

## Discussion

During 2012-2016, the total fatal unintentional occupational injuries registered by ILMO was 8,606. Therefore, every year about 1720 death was registered. In Iranian Social Security Organization database, in 2008 and 2012 only 83 and 96 death registered, respectively^1,6^. During 2008-2012, number of death registered in Ministry of Labor and Social Affairs’ offices database due to occupational injury in Iran was between 24-40 death^8^. Number of fatal occupational injuries in this study was much more than previous study in Iran. One reason for this is the difference in the databases used in previous studies where there are only registered death in insured workers. Mortality can be greater in uninsured populations^9^ and more than 50% of the workers in the world do not have coverage by insurance or Social Security^10^. Our study may also underestimate the occupational deaths due to injuries, because of under-reporting. Some deaths may not be reported, especially in cases of employing illegal workers. Incomplete recording is characteristic of mortality registries, even for insured populations in developed nations. In Australia, about 43% of deaths of all workers at work were underestimated^14^.

In this study, the rate of fatal occupational injuries per 100,000 workers per year was 7.9, and the rate of fatal occupational injuries per 100,000 EAP per year was 6.83 overall. A study estimated the rate of injury mortality at work in the EAP, by WHO sub-region. In Eastern Mediterranean, about 25.5 fatal occupational injuries occurred per 100,000 EAP and this rate was 14.02 in Africa^4^. In Iran, the fatal accidents rate was 0.95 in 100,000^6^. In another study, based on International Labor Organization (ILO) fatal injury estimation, fatal occupational injuries per 100,000 EAP was estimated at 11.7 in Eastern Mediterranean Region^15^. In Turkey, the rate of fatal occupational injury was estimated 22.3 per 100,000 workers^16^. In Korea, the fatal injury rate was 12.59 in 2001 and 8.20 in 2009 per 100,000 workers^17^. The results of the present study compared to other studies done in Iran is closer to estimates. Differences in types of industries, occupational activities, employment characteristics, and implementation of safety measures also explain mortality variability across cities and countries^4,18^. Moreover, the difference between Iran and another country may be partly due to limitations in the quality of information.

In this study, the provinces with the highest rate of YLL are provinces more industrialized. For example, Yazd is one of Iran's industrial centers for textiles. There is also a considerable construction material and ceramics industry. In Hormozgan also, the industries associated with the petroleum refinery, aluminum plant, ship, steel and cement are active. On the other hand, in the provinces with the lowest YLL, the main occupation is agriculture and livestock

Our study showed 98.7% of the deaths occurred in males. A review of ILO data about fatal occupational injury for 21 countries shows that in all countries, between 91% and 99% of all deaths occurred in males, this percent is independent of the level of economic development of the country^4^.

This study showed most victims were 25 to 29 yr old. Death rates from injuries at work vary by age. In most studies, the pattern of data corresponded to a steady rise from the youngest age groups to about 64 year. Only a few countries observed the highest risk in the youngest ages. For example, in the United States, the highest age-specific death rates occurred in 65 yr old worker, whereas in Canada the highest age-specific death rates were observed in workers aged 16-24 yr old^19-21^.

### Limitation

As in many other studies that use existing data, one of the limitations of the present study is under-reporting.

## Conclusion

The analysis of YLL indicated that premature mortality due to occupational injury is still a meaningful problem in the Iranian population. Our findings may be useful from a health policy perspective for designing and prioritizing interventions focused on the prevention of premature loss of life. Known prevention strategies need to be implemented widely to diminish avoidable injuries in the workplace.

## Acknowledgements

Thanks for Legal Medicine Organization for funding.

## Conflict of interest

Not to declare.

## Funding

Legal Medicine Organization supported the study financially.

## Highlights

- Premature mortality due to occupational injury is an important problem in Iran.
- In 2012-2016, unintentional occupational injuries caused 8,606 deaths in Iran.
- The rate of YLL due to occupational injury was 3.99 per 1,000 workers per year.
- Almost 98.7% of the fatal unintentional occupational injuries occurred in males.
